# Supplementary material for: Microbial Transformation of Prenylquercetins by Mucor hiemalis
Source: Molecules. 2020 Jan 25;25(3):528. doi: 10.3390/molecules25030528 (PMC7037548; doi:10.3390/molecules25030528)
Supplement: Supplementary file 1 [file molecules-25-00528-s001.pdf]

Supplementary Materials

Microbial Transformation of Prenylquercetins by *Mucor hiemalis*

Fubo Han, Yina Xiao and Ik-Soo Lee*

College of Pharmacy, Chonnam National University, Gwangju 61186, Korea;
hanfubo0306@gmail.com (F.H.); yogurtxiao@163.com (Y.X.)
* Correspondence: islee@chonnam.ac.kr; Tel.: +82-62-530-2932

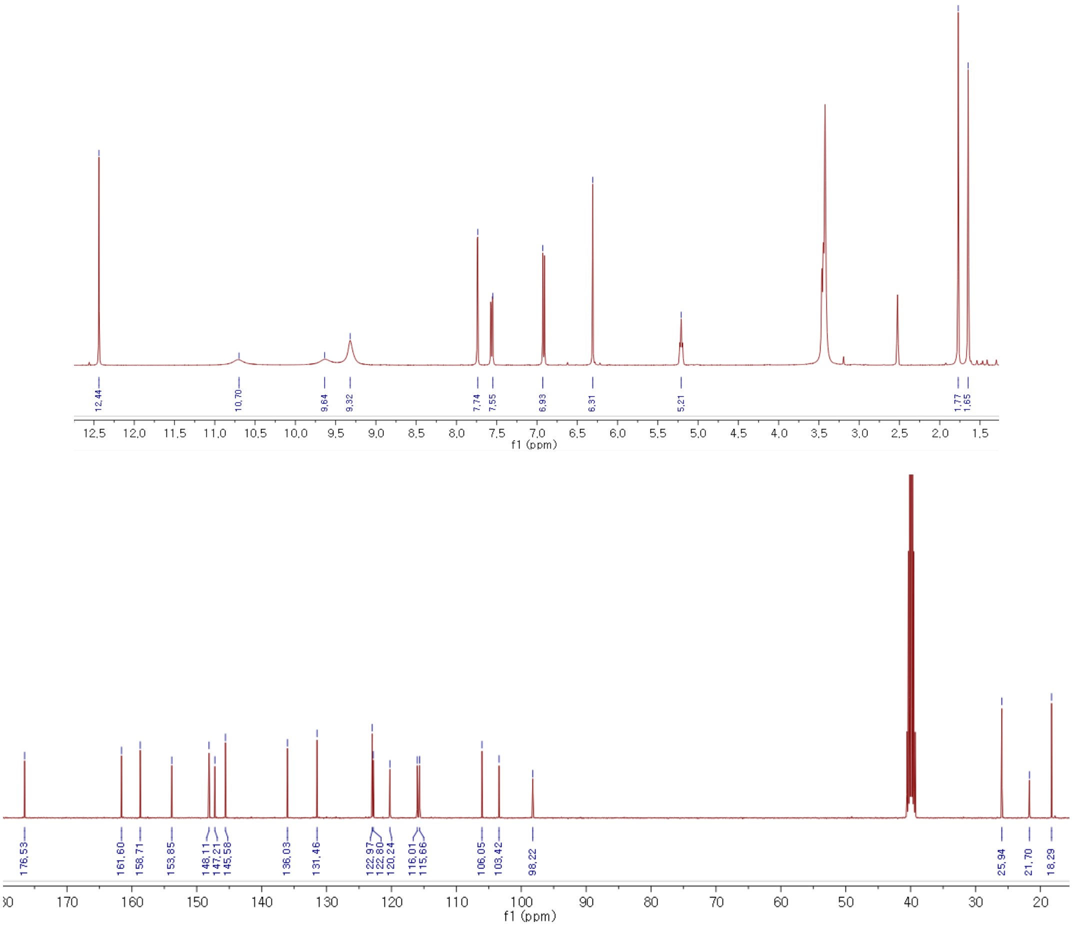

Figure S1. ^{1}H and ^{13}C NMR spectra of 8-prenylquercetin (**1**) (DMSO-d_6)

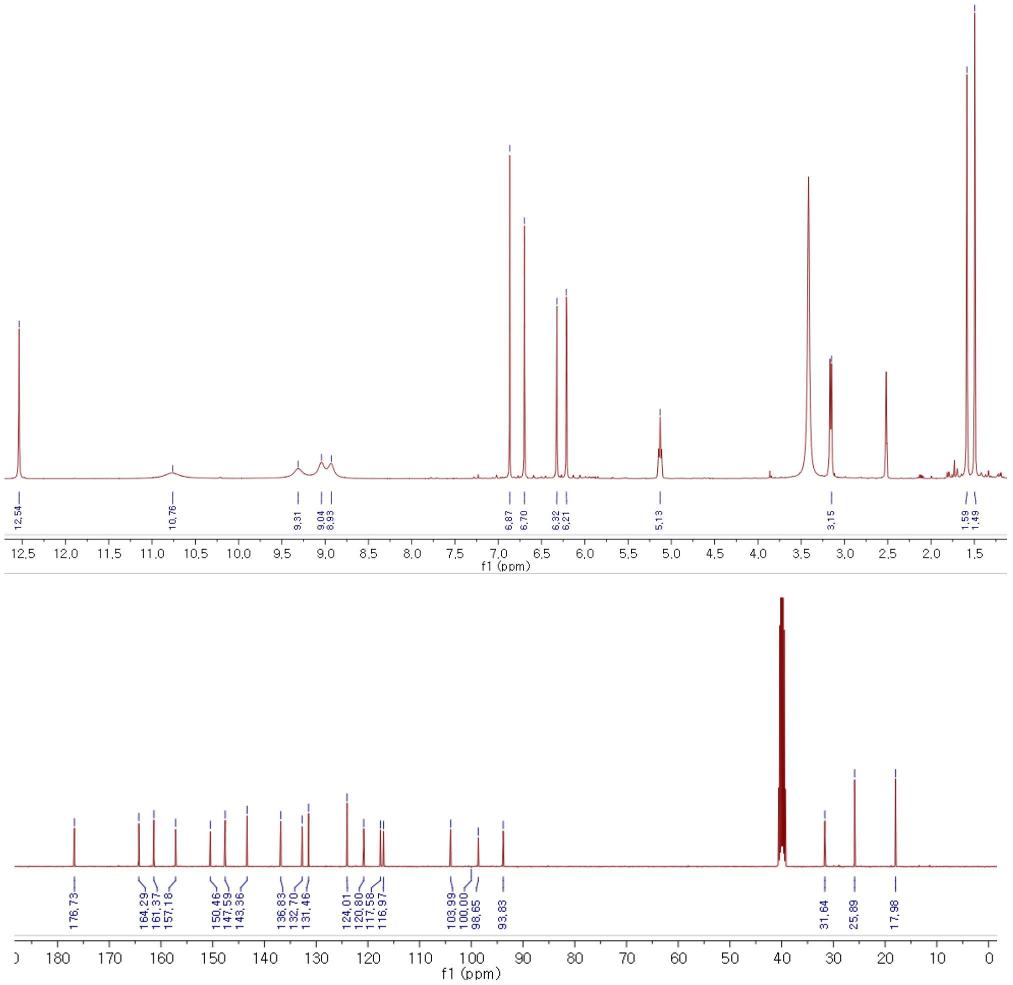

Figure S2. ^{1}H and ^{13}C NMR spectra of 6′-prenylquercetin (**2**) (DMSO-d_6)

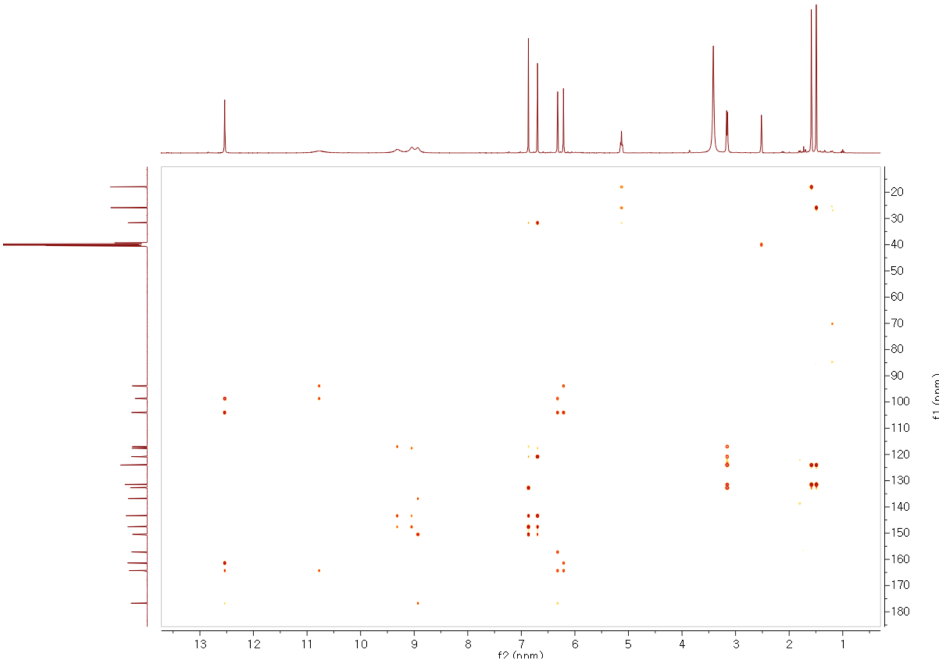

Figure S3. HMBC spectrum of 6′-prenylquercetin (**2**) (DMSO-d_6)

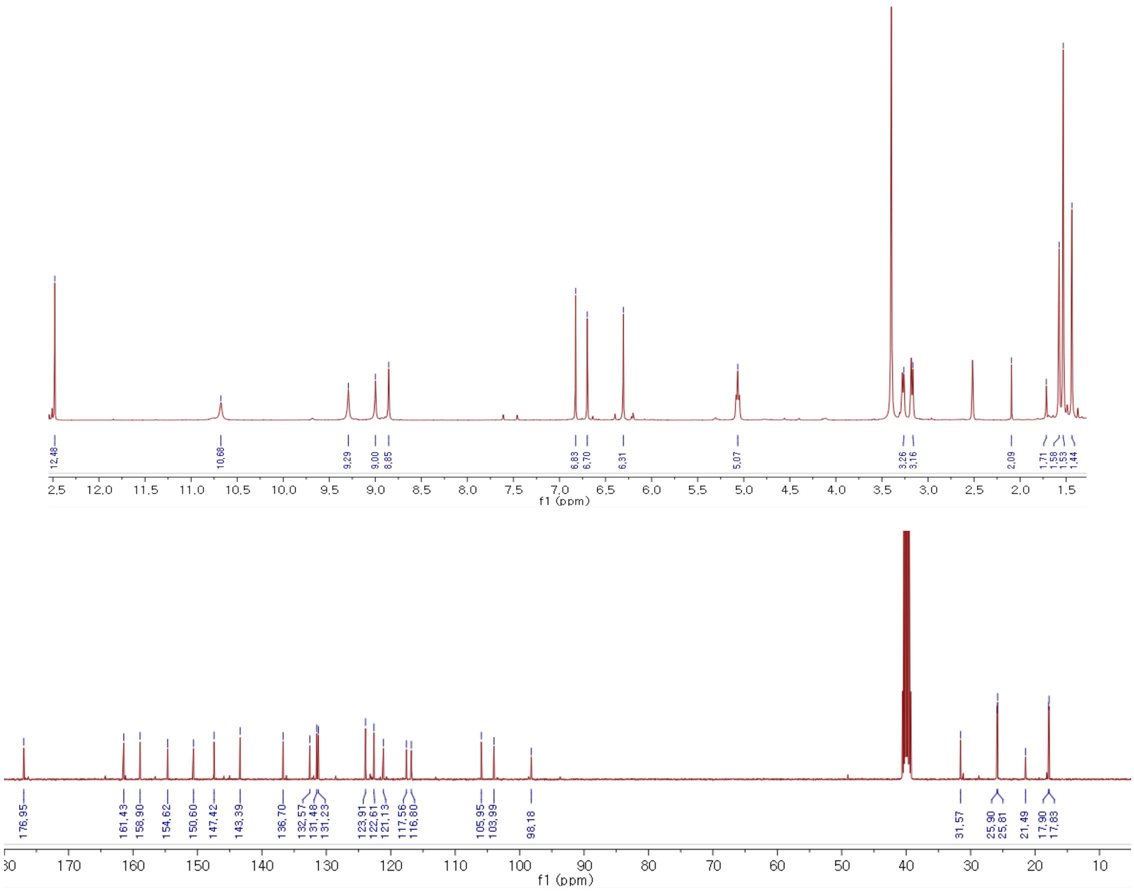

Figure S4. ^{1}H and ^{13}C NMR spectra of 8,6′-diprenylquercetin (**3**) (DMSO-d_6)

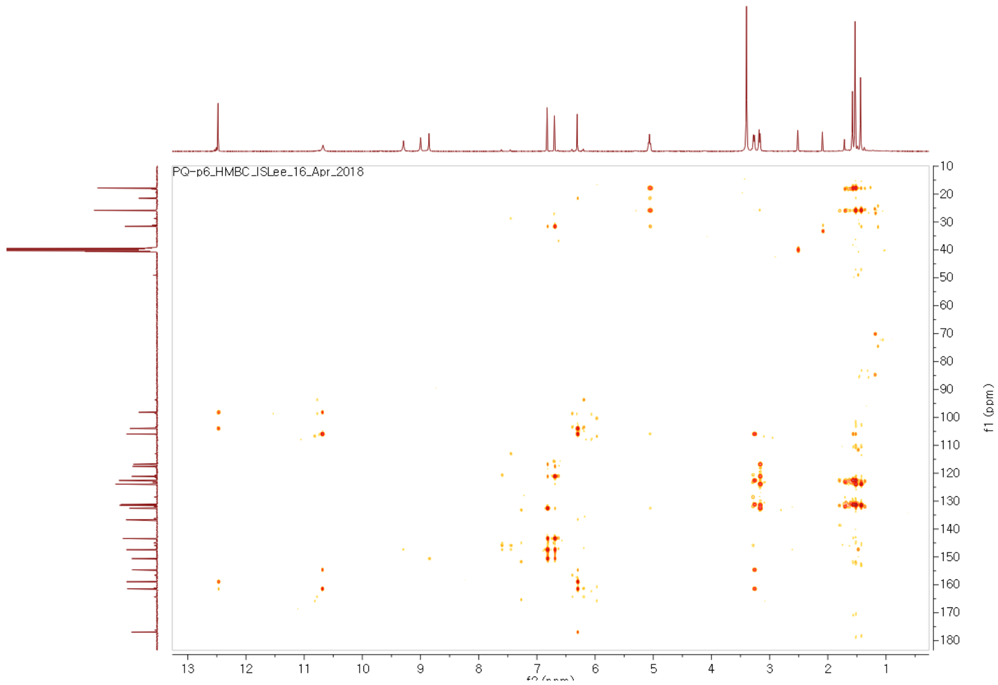

Figure S5. HMBC spectrum of 8,6′-diprenylquercetin (**3**) (DMSO-d_6)

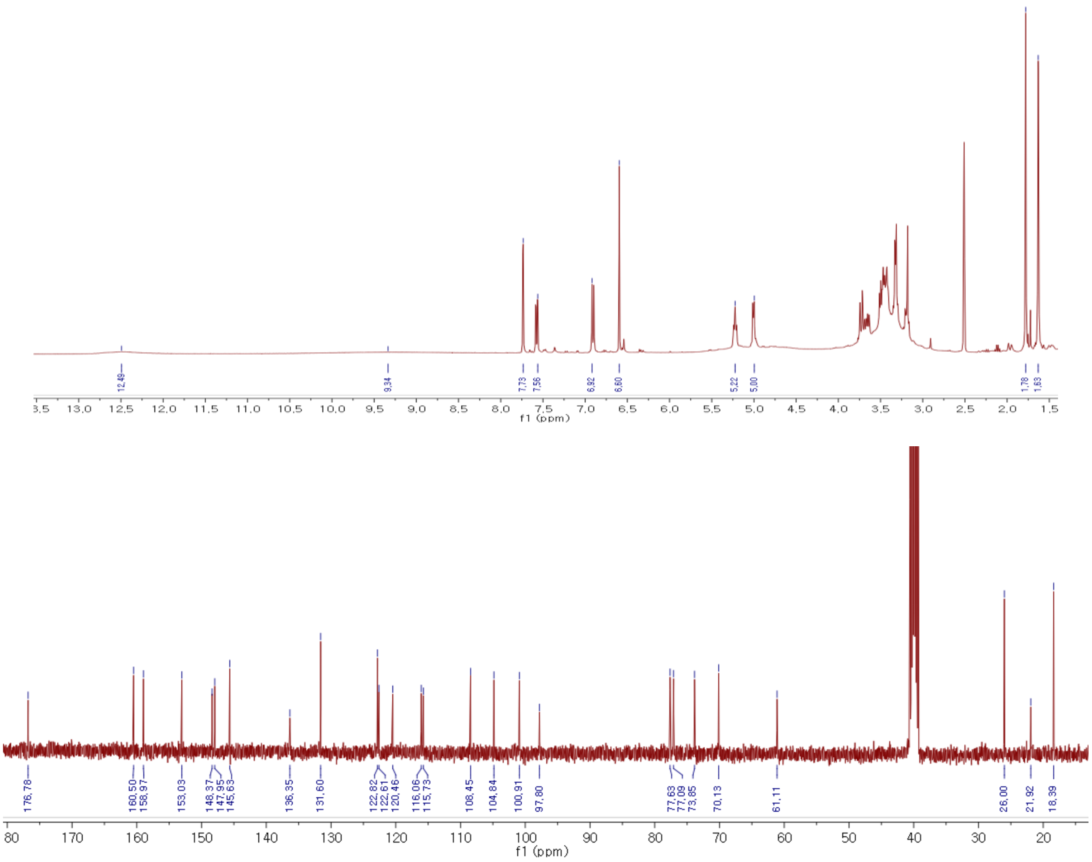

Figure S6. ^{1}H and ^{13}C NMR spectra of metabolite **4** (DMSO-d_6)

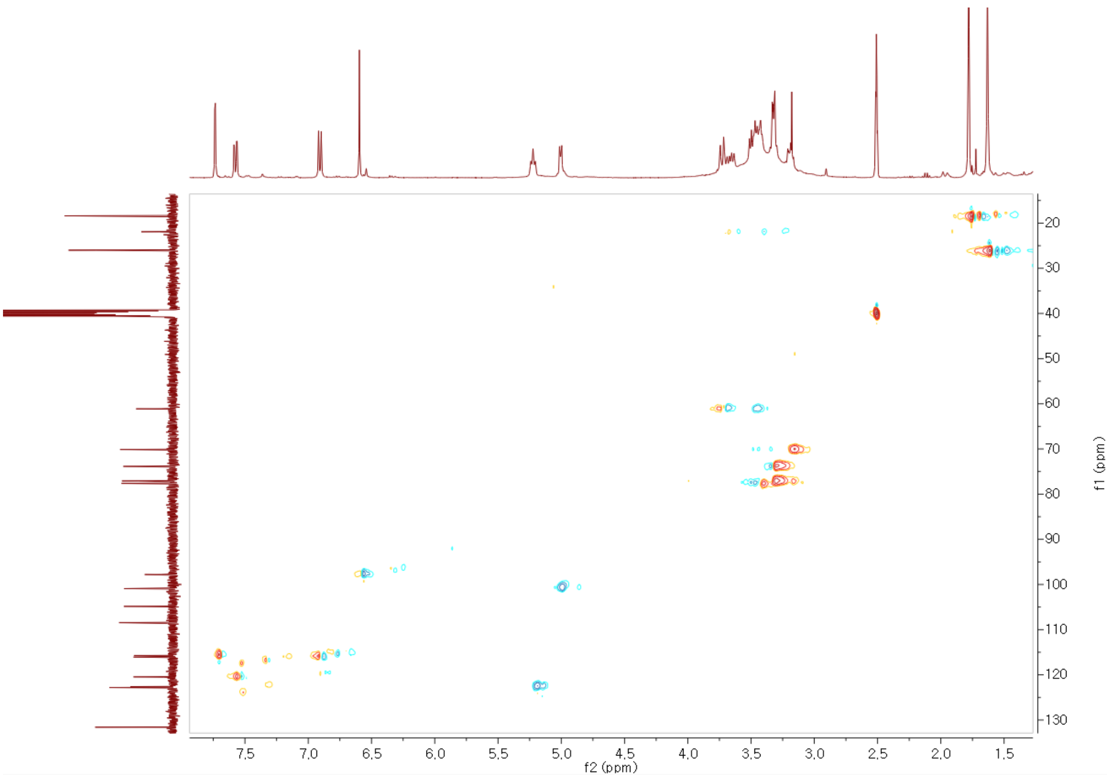

Figure S7. HSQC spectrum of metabolite **4** (DMSO-d_6)

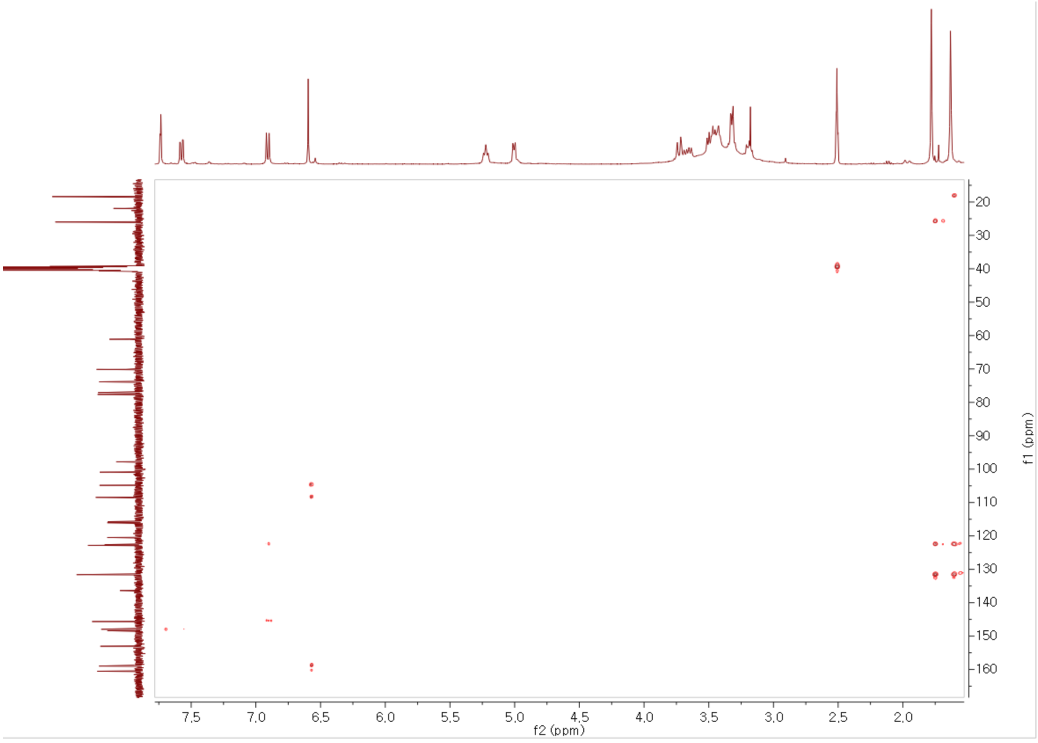

Figure S8. HMBC spectrum of metabolite **4** (DMSO-d_6)

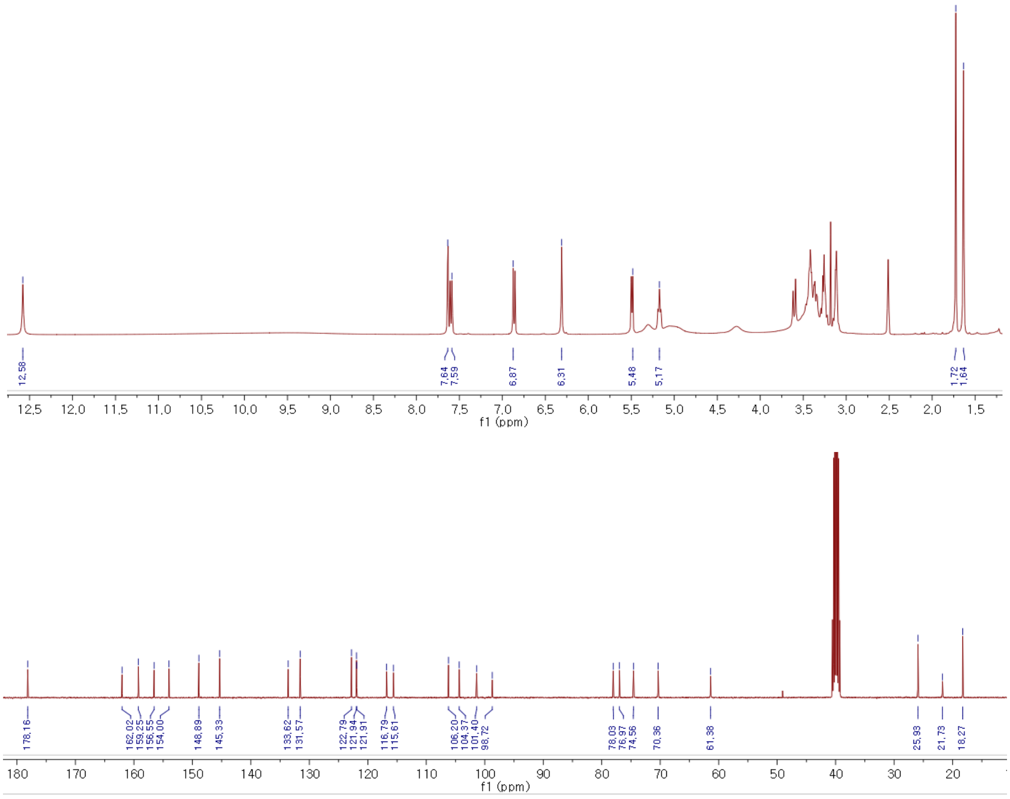

Figure S9. ^{1}H and ^{13}C NMR spectra of metabolite **5** (DMSO-d_6)

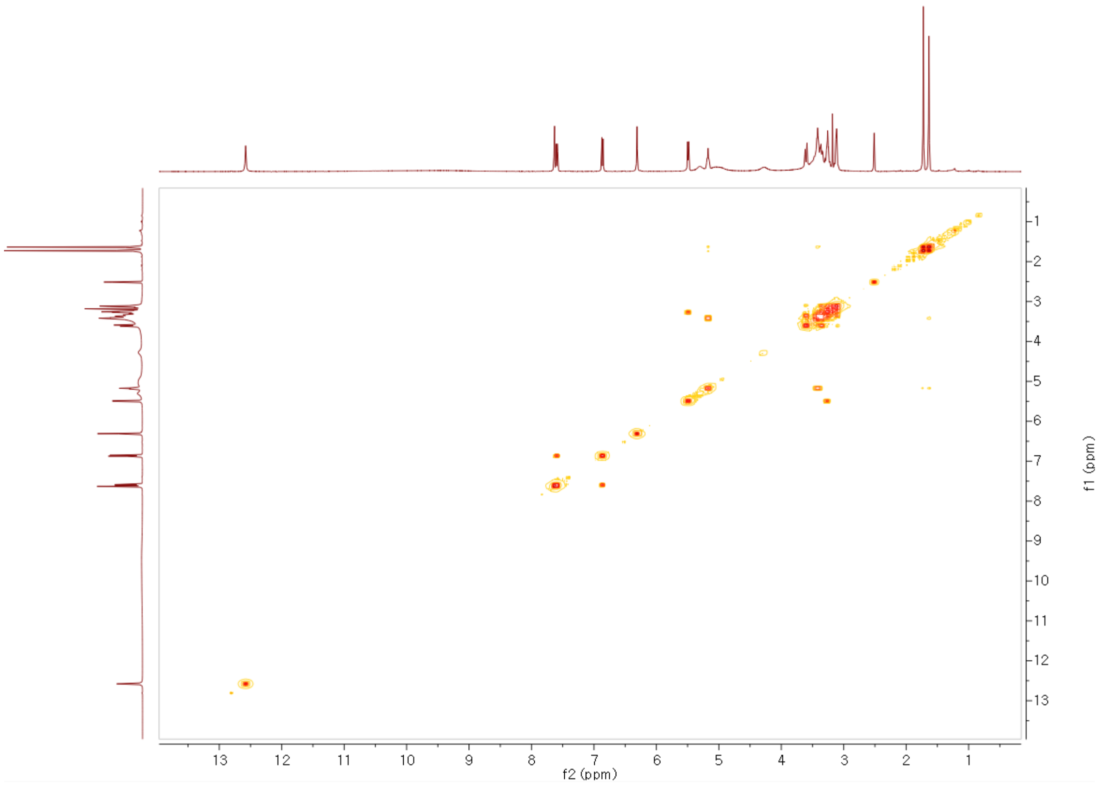

Figure S10. COSY spectrum of metabolite **5** (DMSO-d_6)

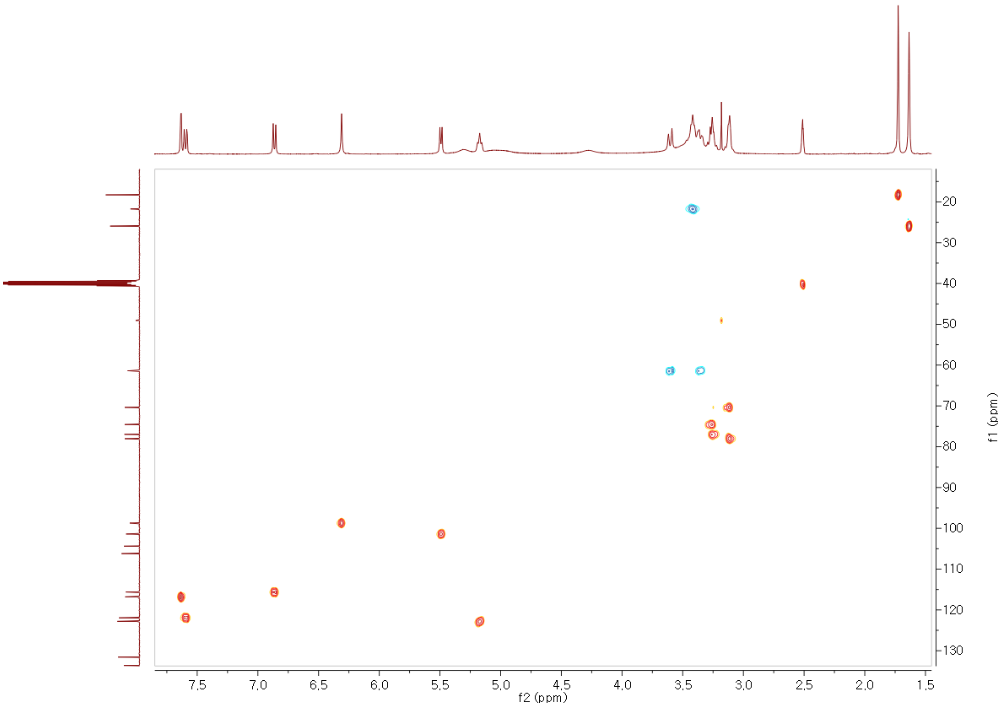

Figure S11. HSQC spectrum of metabolite **5** (DMSO-d_6)

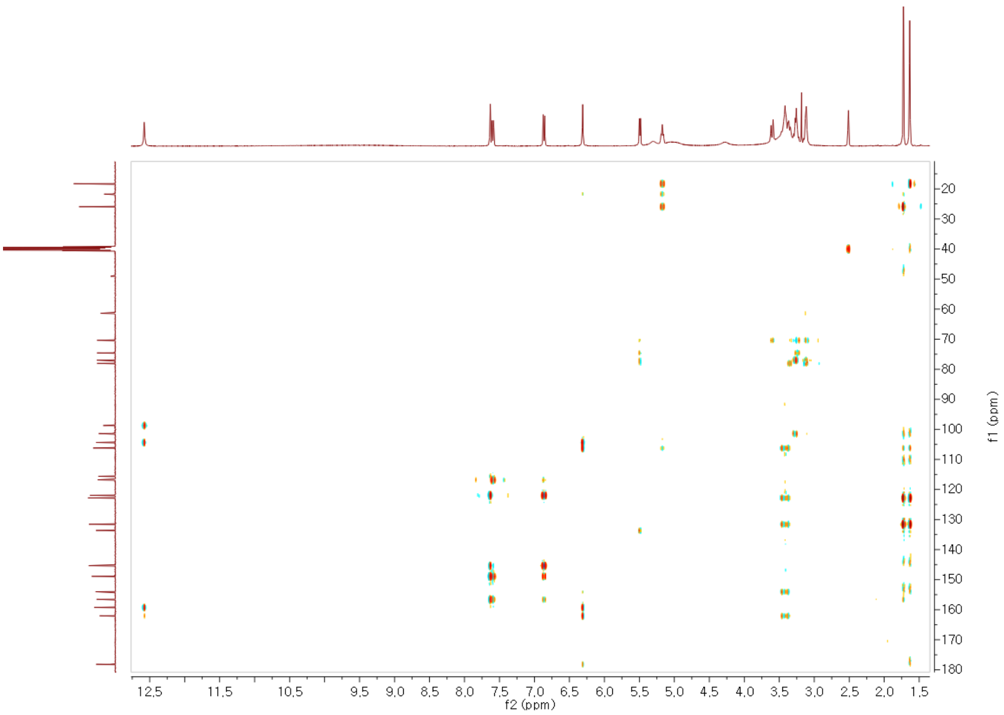

Figure S12. HMBC spectrum of metabolite **5** (DMSO-d_6)

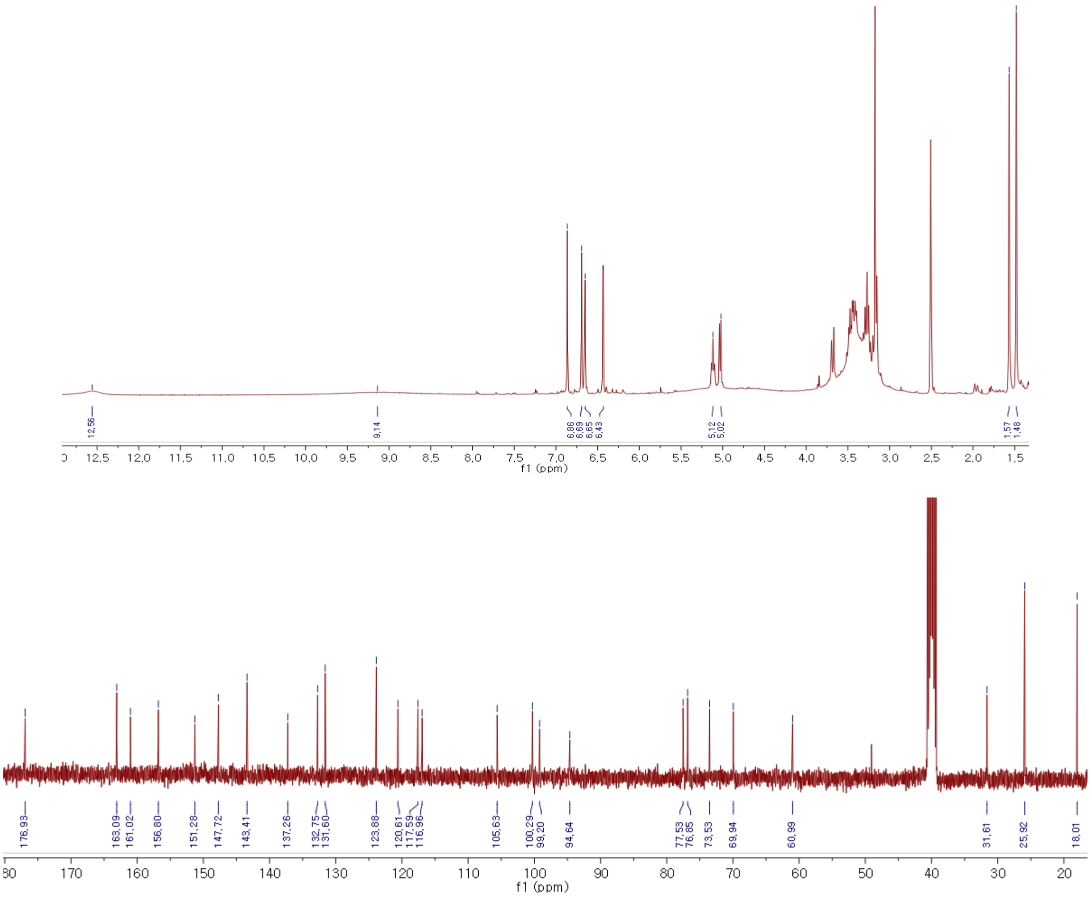

Figure S13. ^{1}H and ^{13}C NMR spectra of metabolite **6** (DMSO-d_6)

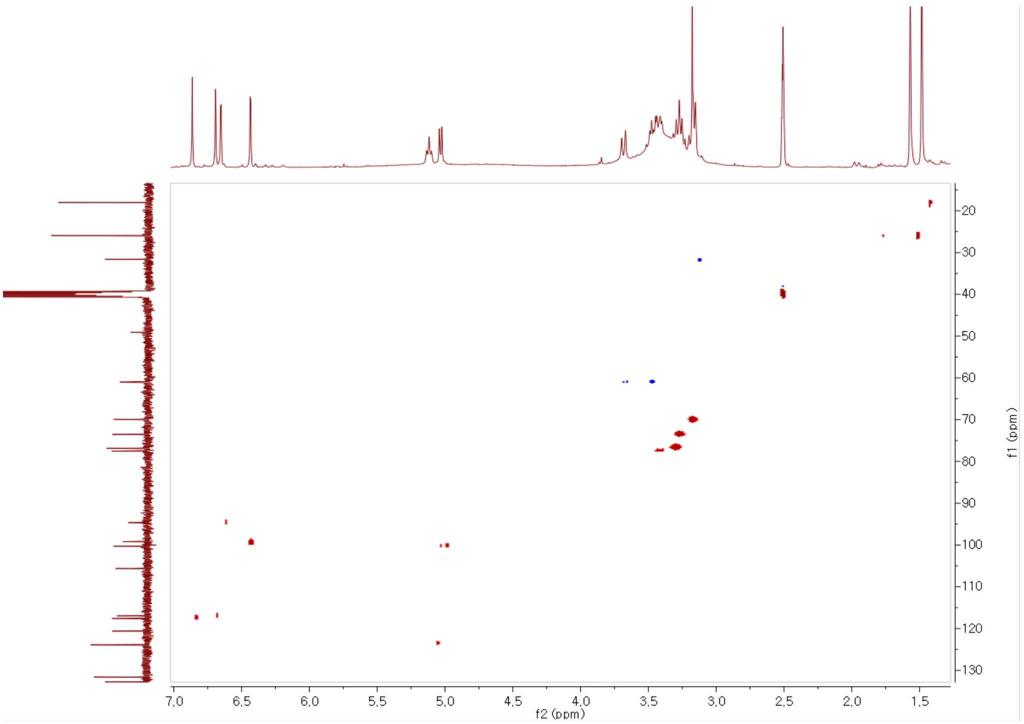

Figure S14. HSQC spectrum of metabolite **6** (DMSO-d_6)

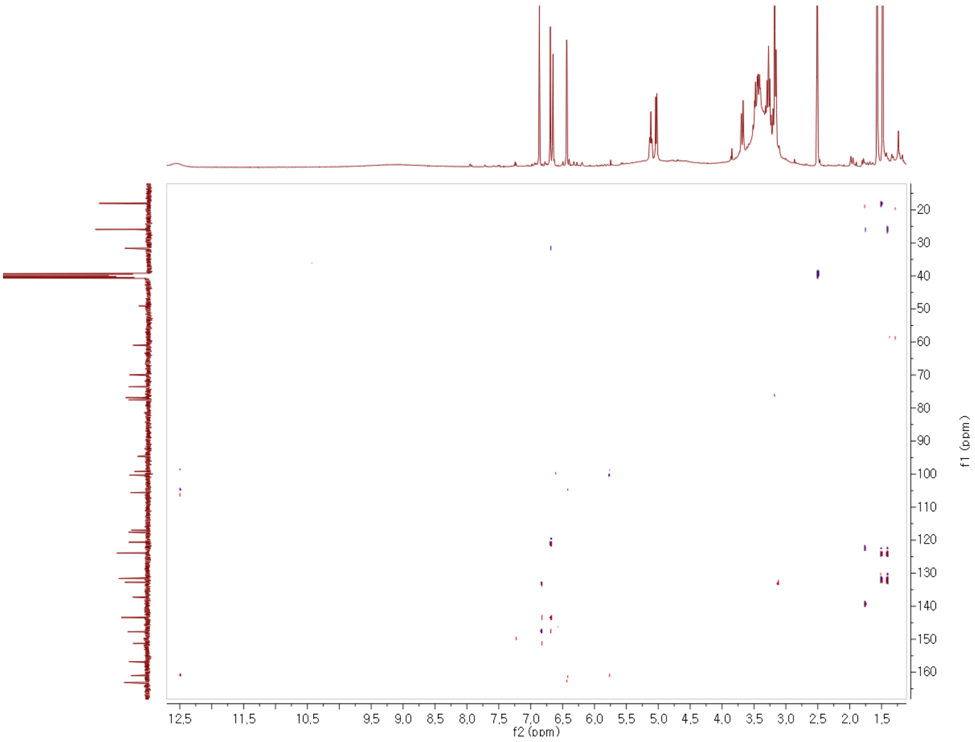

Figure S15. HMBC spectrum of metabolite **6** (DMSO-d_6)

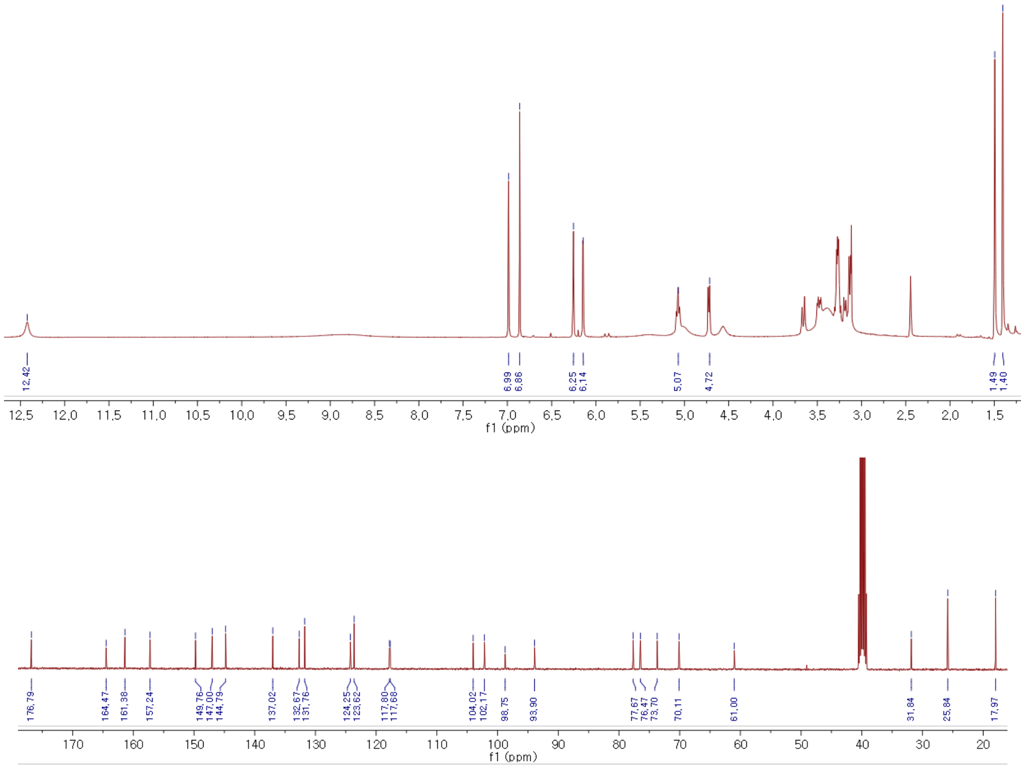

Figure S16. ^{1}H and ^{13}C NMR spectra of metabolite **7** (DMSO-d_6)

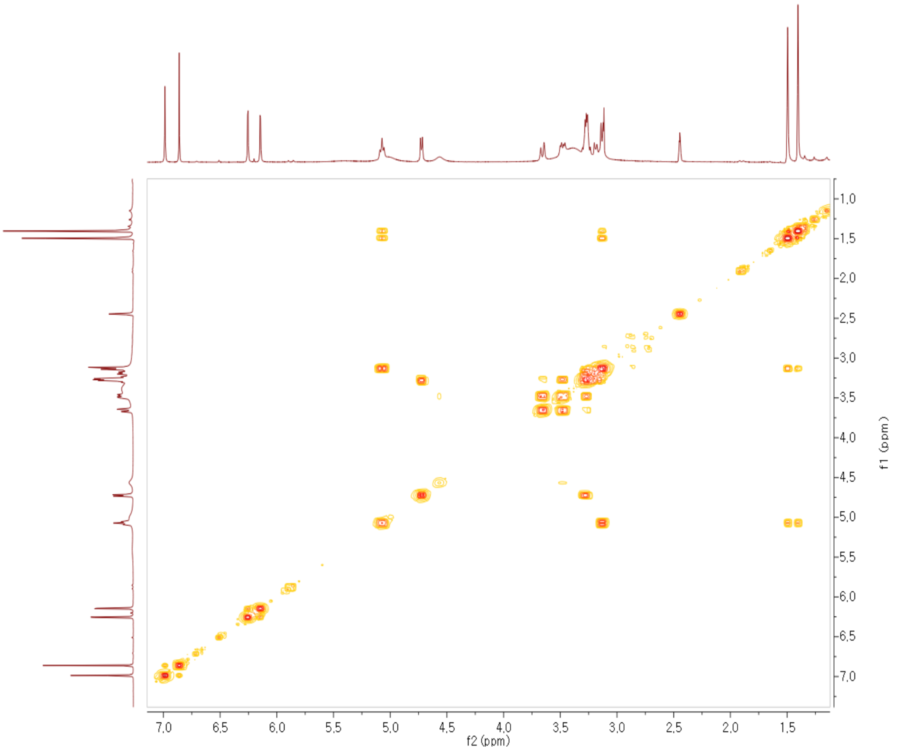

Figure S17. COSY spectrum of metabolite **7** (DMSO-d_6)

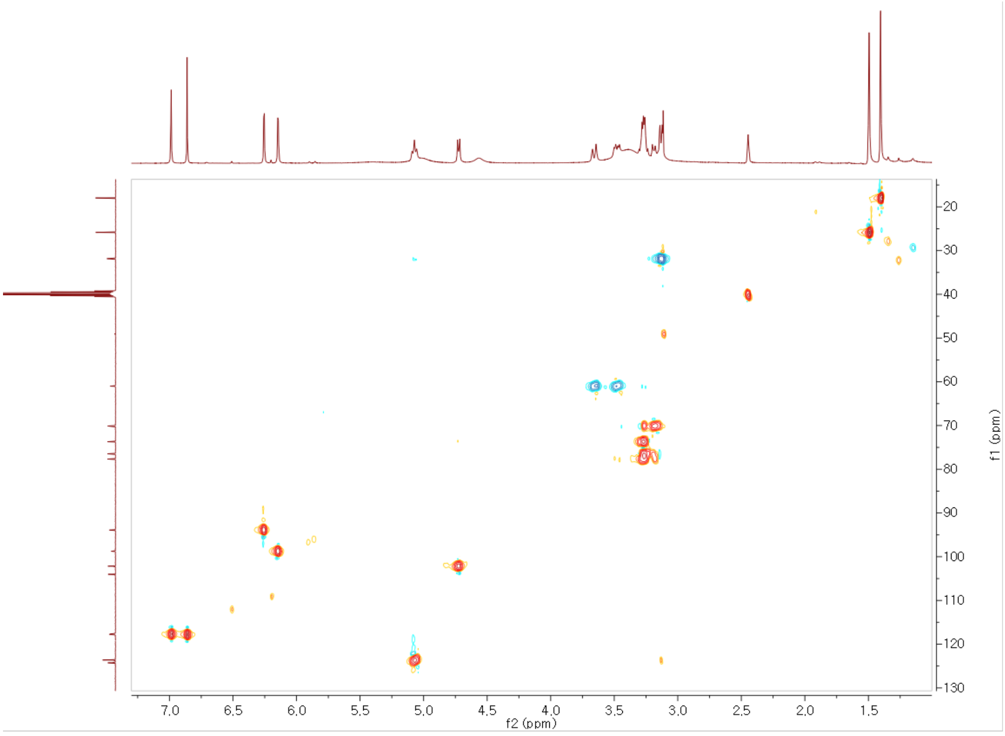

Figure S18. HSQC spectrum of metabolite **7** (DMSO-d_6)

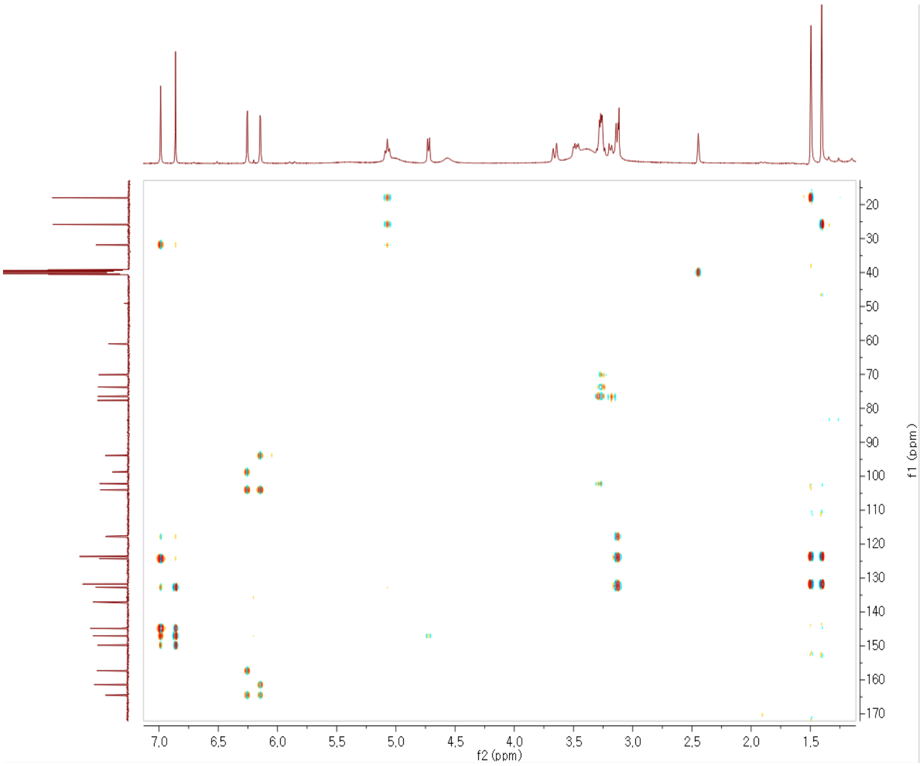

Figure S19. HMBC spectrum of metabolite **7** (DMSO-d_6)

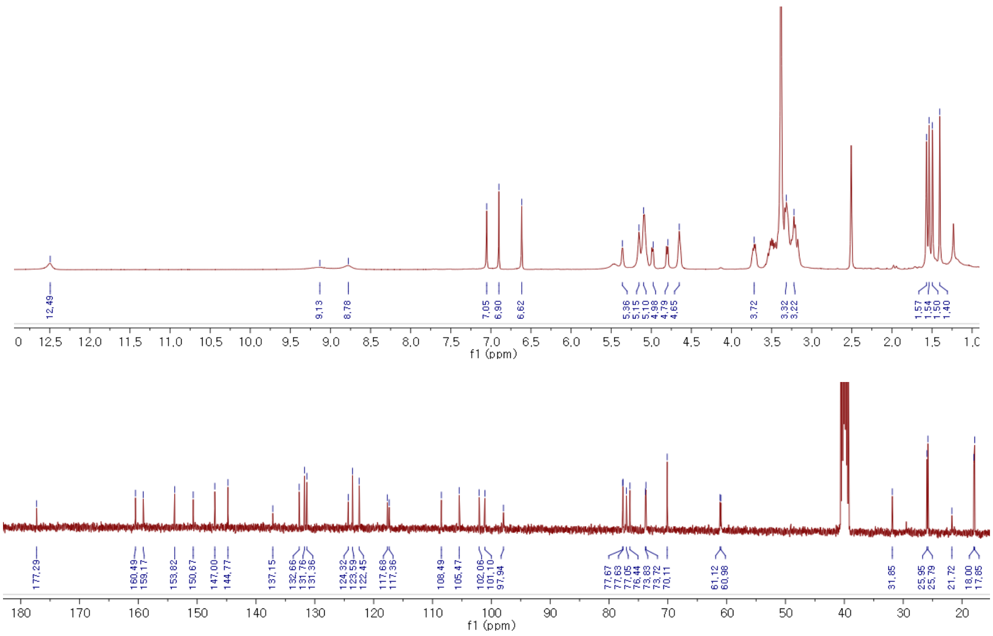

Figure S20. ^{1}H and ^{13}C NMR spectra of metabolite **8** (DMSO-d_6)

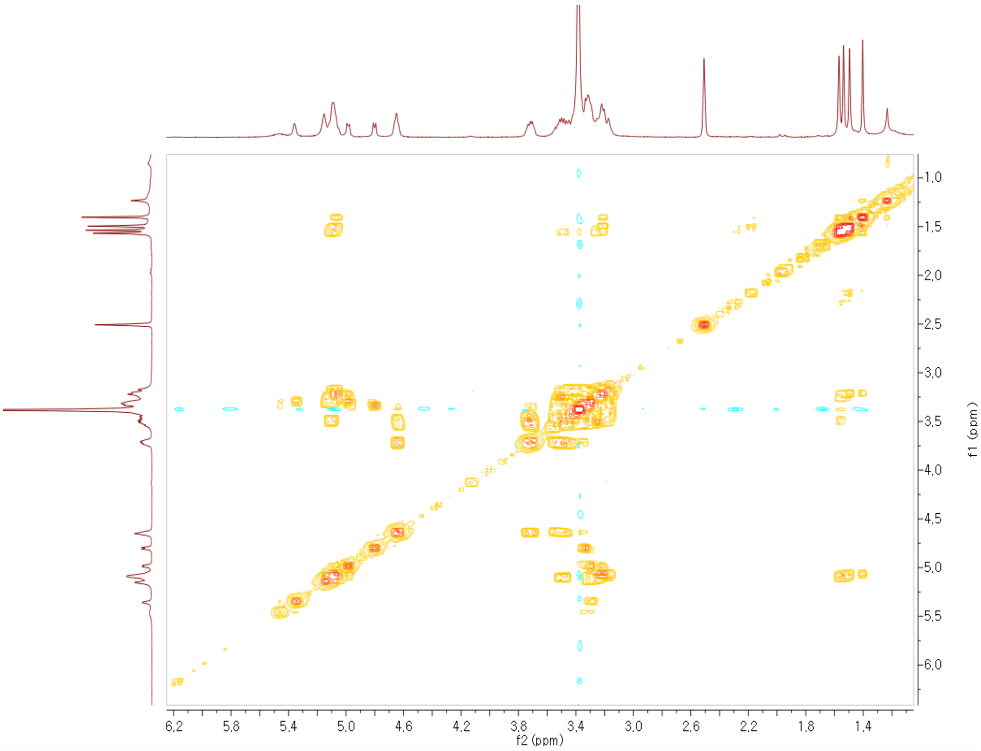

Figure S21. COSY spectrum of metabolite **8** (DMSO-d_6)

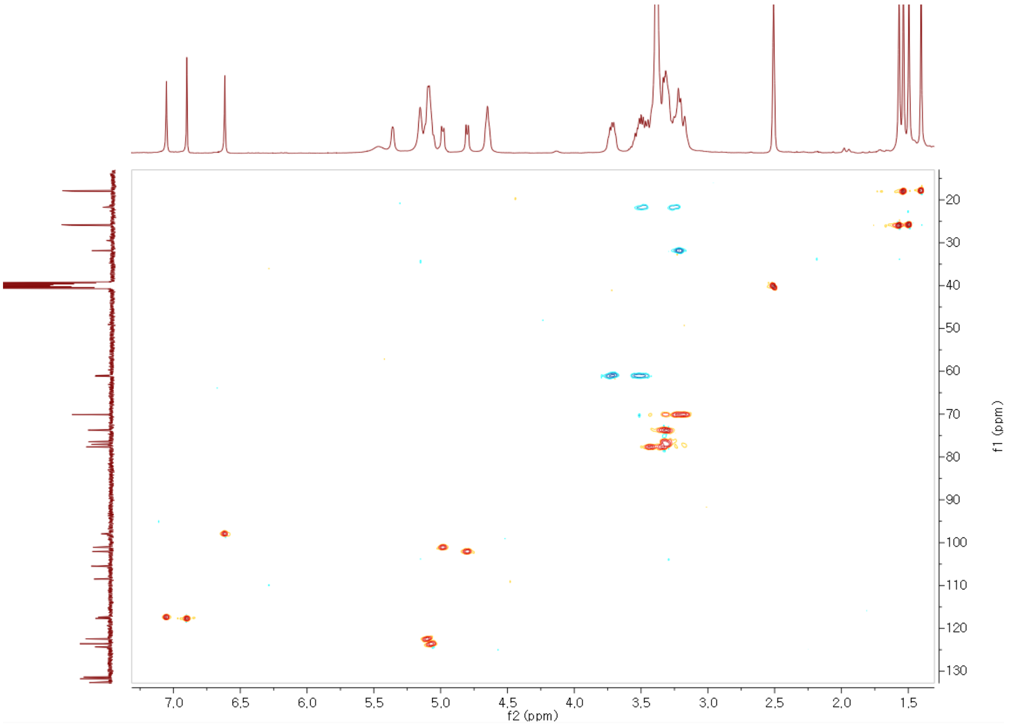

Figure S22. HSQC spectrum of metabolite **8** (DMSO-d_6)

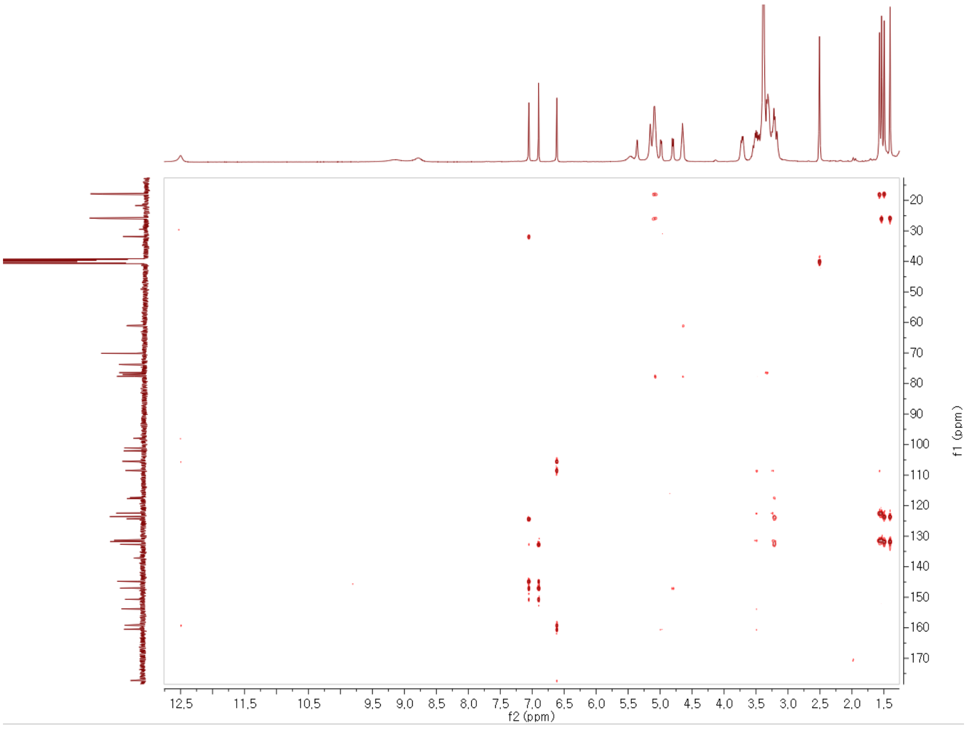

Figure S23. HMBC spectrum of metabolite **8** (DMSO-d_6)

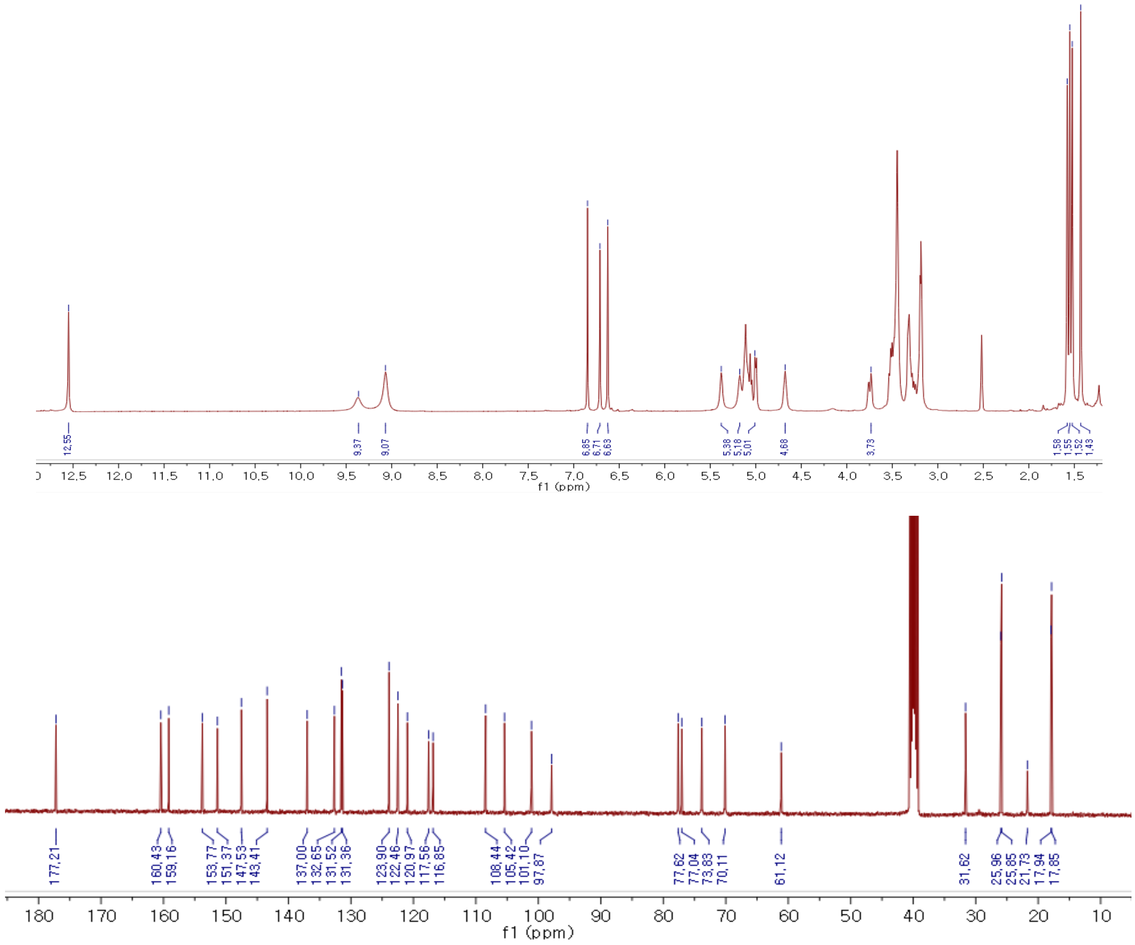

Figure S24. ^{1}H and ^{13}C NMR spectra of metabolite **9** (DMSO-d_6)

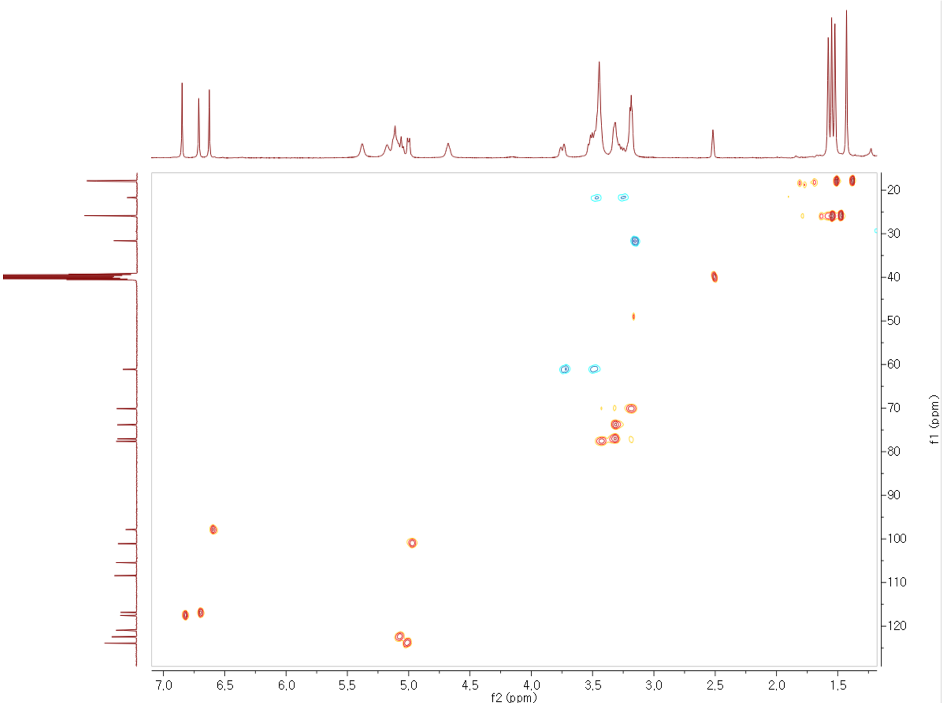

Figure S25. HSQC spectrum of metabolite **9** (DMSO-d_6)

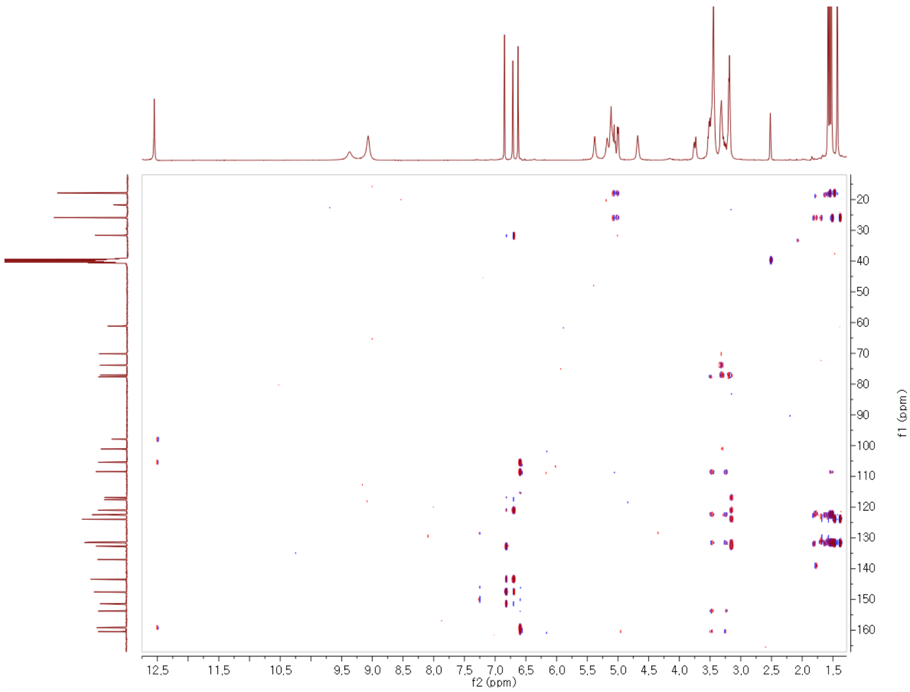

Figure S26. HMBC spectrum of metabolite **9** (DMSO-d_6)

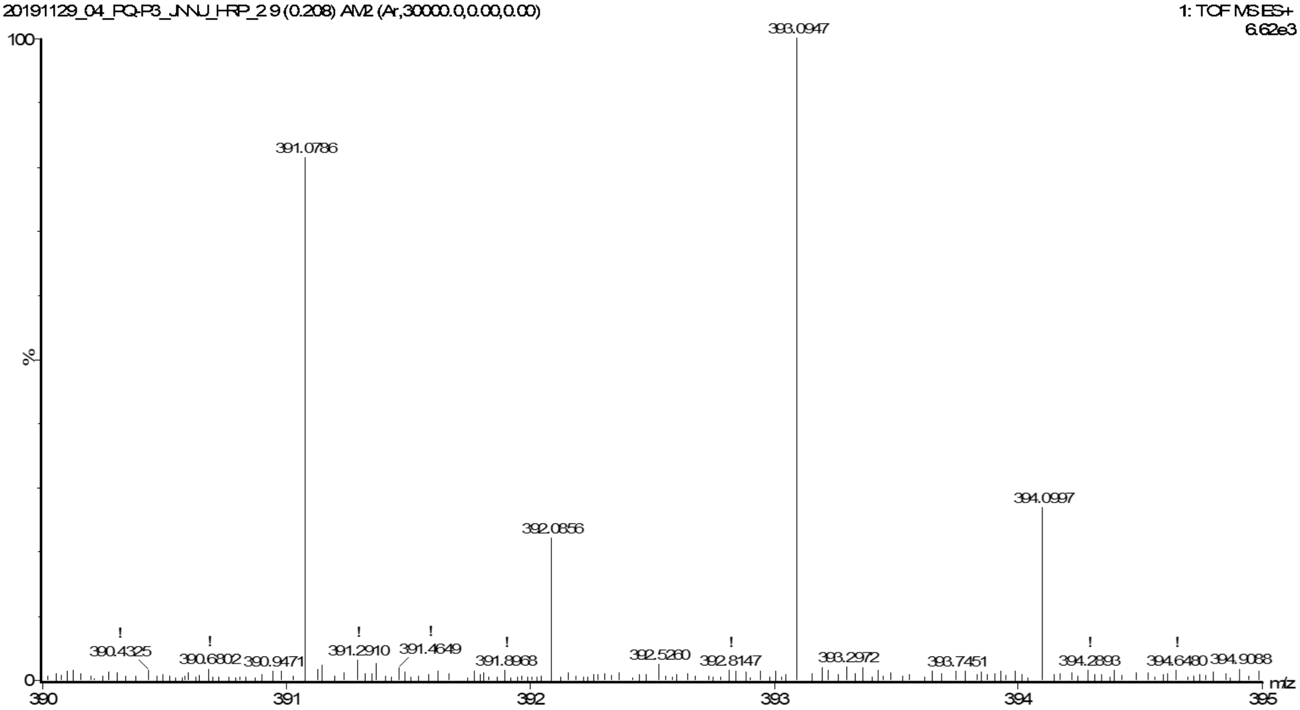

Elemental Composition Report

Single Mass Analysis
Tolerance = 5.0 PPM / DBE: min = -1.5, max = 100.0
Element prediction: Off
Number of isotope peaks used for i-FIT = 3

Monoisotopic Mass, Even Electron Ions
132 formula(e) evaluated with 1 results within limits (up to 100 closest results for each mass)
Elements Used:
C: 1-40 H: 1-50 O: 1-20 Na: 0-1

Minimum:				-1.5				
Maximum:		100.0	5.0	100.0				
Mass	Calc. Mass	mDa	PPM	DBE	i-FIT	Norm	Conf(%)	Formula
393.0947	393.0950	-0.3	-0.8	11.5	590.6	n/a	n/a	C20 H18 O7 Na

Figure S27. HRESIMS spectrum of compound **2**

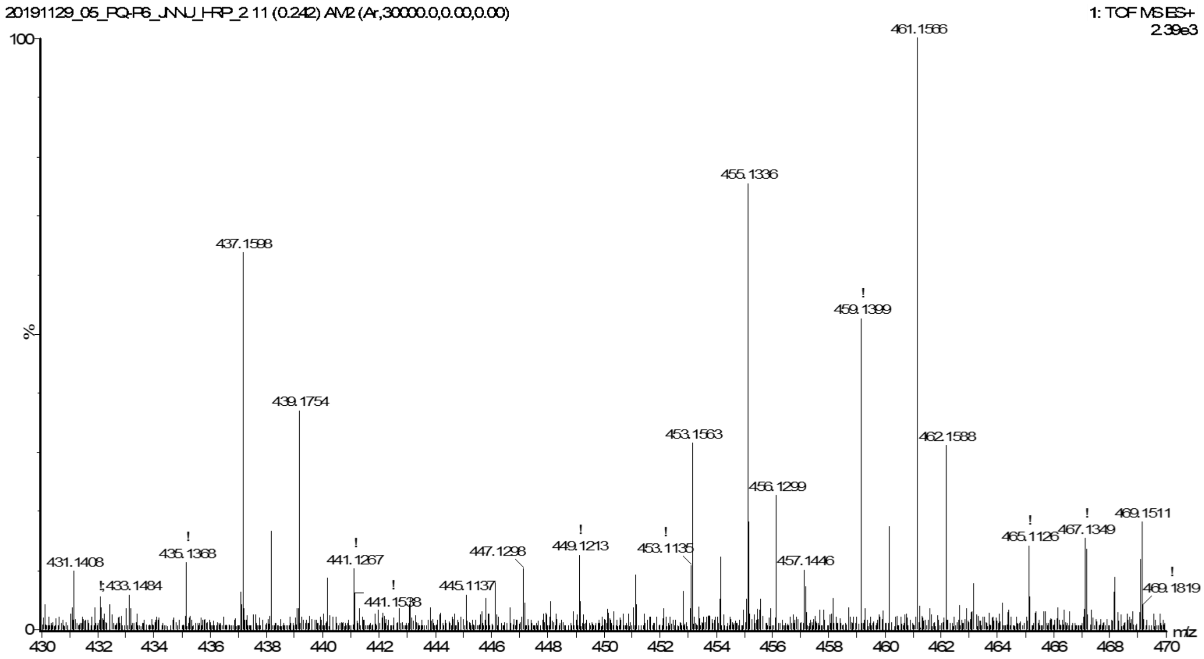

Elemental Composition Report

Single Mass Analysis
Tolerance = 5.0 PPM / DBE: min = -1.5, max = 100.0
Element prediction: Off
Number of isotope peaks used for i-FIT = 3

Monoisotopic Mass, Even Electron Ions
Elements Used:
C: 1-40 H: 1-50 O: 1-20 Na: 0-1

Mass	Calc. Mass	mDa	PPM	DBE	i-FIT	Norm	Conf(%)	Formula
Minimum:				-1.5				
Maximum:		100.0	5.0	100.0				
439.1754	439.1757	-0.3	-0.7	12.5	402.9	1.602	20.15	C25 H27 O7
	439.1733	2.1	4.8	9.5	401.5	0.225	79.85	C23 H28 O7 Na
461.1566	461.1576	-1.0	-2.2	12.5	444.0	n/a	n/a	C25 H26 O7 Na

Figure S28. HRESIMS spectrum of compound **3**

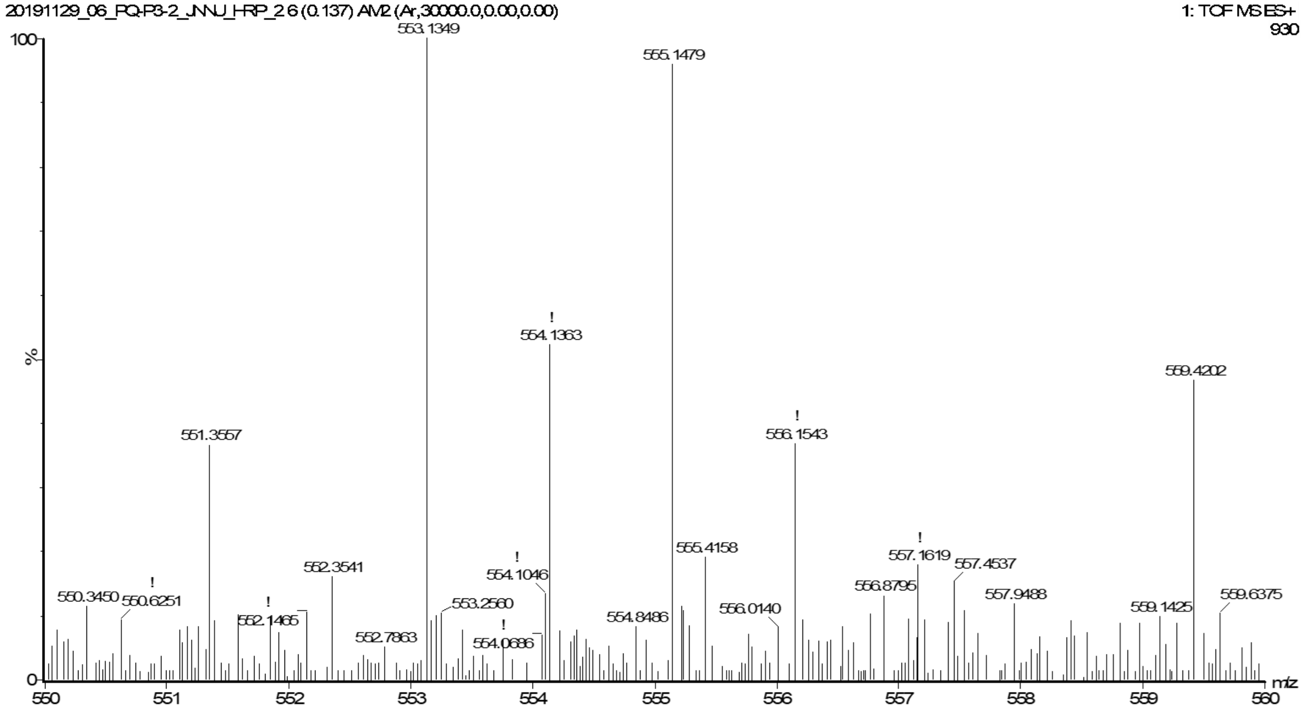

Elemental Composition Report

Single Mass Analysis
Tolerance = 5.0 PPM / DBE: min = -1.5, max = 100.0
Element prediction: Off
Number of isotope peaks used for i-FIT = 3

Monoisotopic Mass, Even Electron Ions
145 formula(e) evaluated with 2 results within limits (up to 100 closest results for each mass)
Elements Used:
C: 1-40 H: 1-50 O: 1-20 Na: 0-1

Mass	Calc. Mass	mDa	PPM	DBE	i-FIT	Norm	Conf(%)	Formula
Minimum:				-1.5				
Maximum:		100.0	5.0	100.0				
555.1479	555.1478	0.1	0.2	12.5	409.0	1.109	32.99	C26 H28 O12 Na
	555.1503	-2.4	-4.3	15.5	408.3	0.400	67.01	C28 H27 O12

Figure S29. HRESIMS spectrum of metabolite **4**

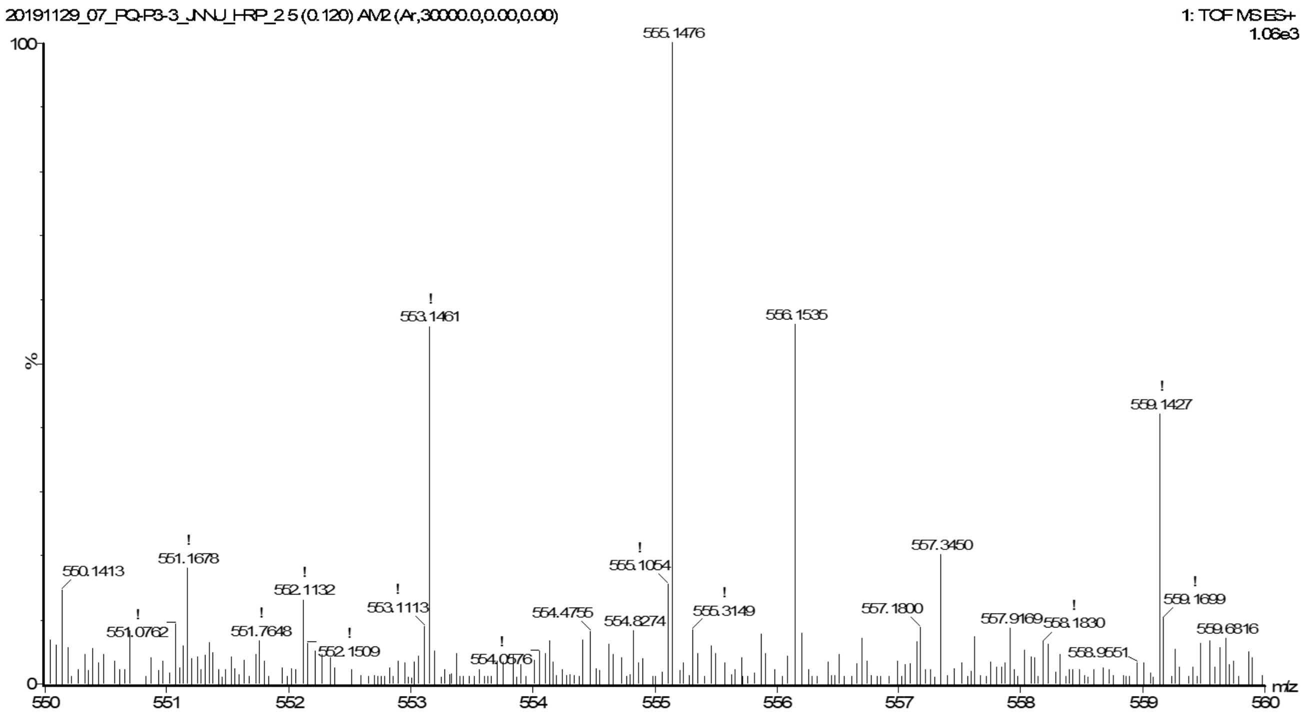

Elemental Composition Report

Single Mass Analysis
Tolerance = 5.0 PPM / DBE: min = -1.5, max = 100.0
Element prediction: Off
Number of isotope peaks used for i-FIT = 3

Monoisotopic Mass, Even Electron Ions
145 formula(e) evaluated with 2 results within limits (up to 100 closest results for each mass)
Elements Used:
C: 1-40 H: 1-50 O: 1-20 Na: 0-1

Mass	Calc. Mass	mDa	PPM	DBE	i-FIT	Norm	Conf(%)	Formula
Minimum:				-1.5				
Maximum:		100.0	5.0	100.0				
555.1476	555.1478	-0.2	-0.4	12.5	355.6	0.714	48.97	C26 H28 O12 Na
	555.1503	-2.7	-4.9	15.5	355.6	0.673	51.03	C28 H27 O12

Figure S30. HRESIMS spectrum of metabolite **5**

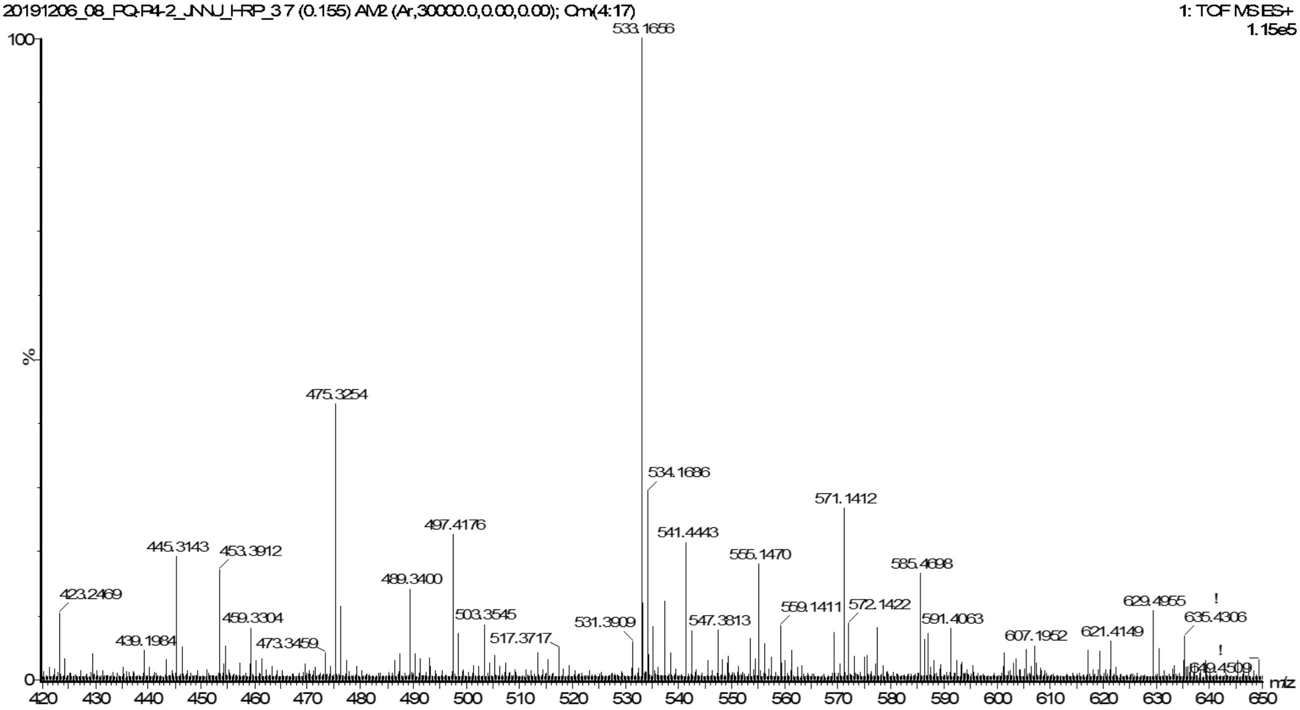

Elemental Composition Report

Single Mass Analysis
Tolerance = 5.0 PPM / DBE: min = -1.5, max = 100.0
Element prediction: Off
Number of isotope peaks used for i-FIT = 3

Monoisotopic Mass, Even Electron Ions
48 formula(e) evaluated with 2 results within limits (up to 100 closest results for each mass)
Elements Used:
C: 1-30 H: 1-30 O: 0-15 Na: 0-1

Minimum:				-1.5				
Maximum:		100.0	5.0	100.0				
Mass	Calc. Mass	mDa	PPM	DBE	i-FIT	Norm	Conf(%)	Formula
533.1656	533.1659	-0.3	-0.6	12.5	748.3	0.028	97.24	C26 H29 O12
	533.1635	2.1	3.9	9.5	751.8	3.591	2.76	C24 H30 O12 Na

Figure S31. HRESIMS spectrum of metabolite **6**

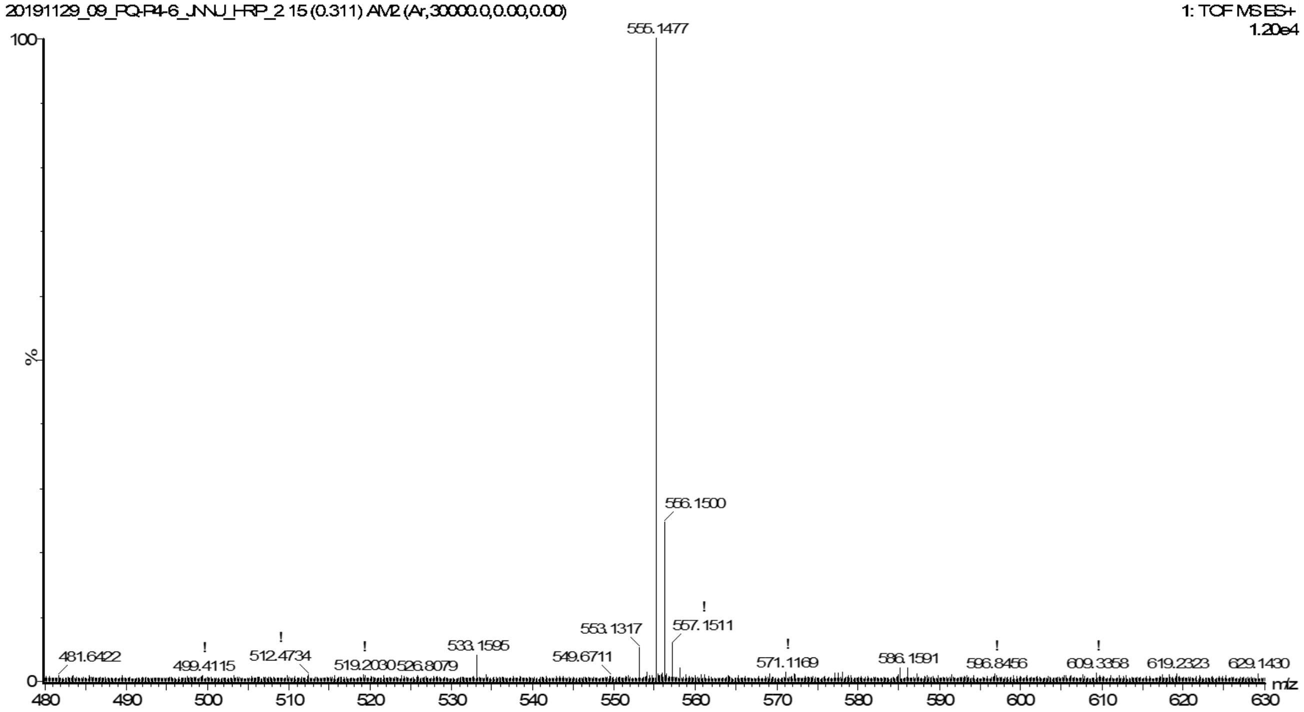

Elemental Composition Report

Single Mass Analysis
Tolerance = 5.0 PPM / DBE: min = -1.5, max = 100.0
Element prediction: Off
Number of isotope peaks used for i-FIT = 3

Monoisotopic Mass, Even Electron Ions
145 formula(e) evaluated with 2 results within limits (up to 100 closest results for each mass)
Elements Used:
C: 1-40 H: 1-50 O: 1-20 Na: 0-1

Minimum:				-1.5				
Maximum:		100.0	5.0	100.0				
Mass	Calc. Mass	mDa	PPM	DBE	i-FIT	Norm	Conf(%)	Formula
555.1477	555.1478	-0.1	-0.2	12.5	437.2	0.042	95.87	C26 H28 O12 Na
	555.1503	-2.6	-4.7	15.5	440.3	3.187	4.13	C28 H27 O12

Figure S32. HRESIMS spectrum of metabolite **7**

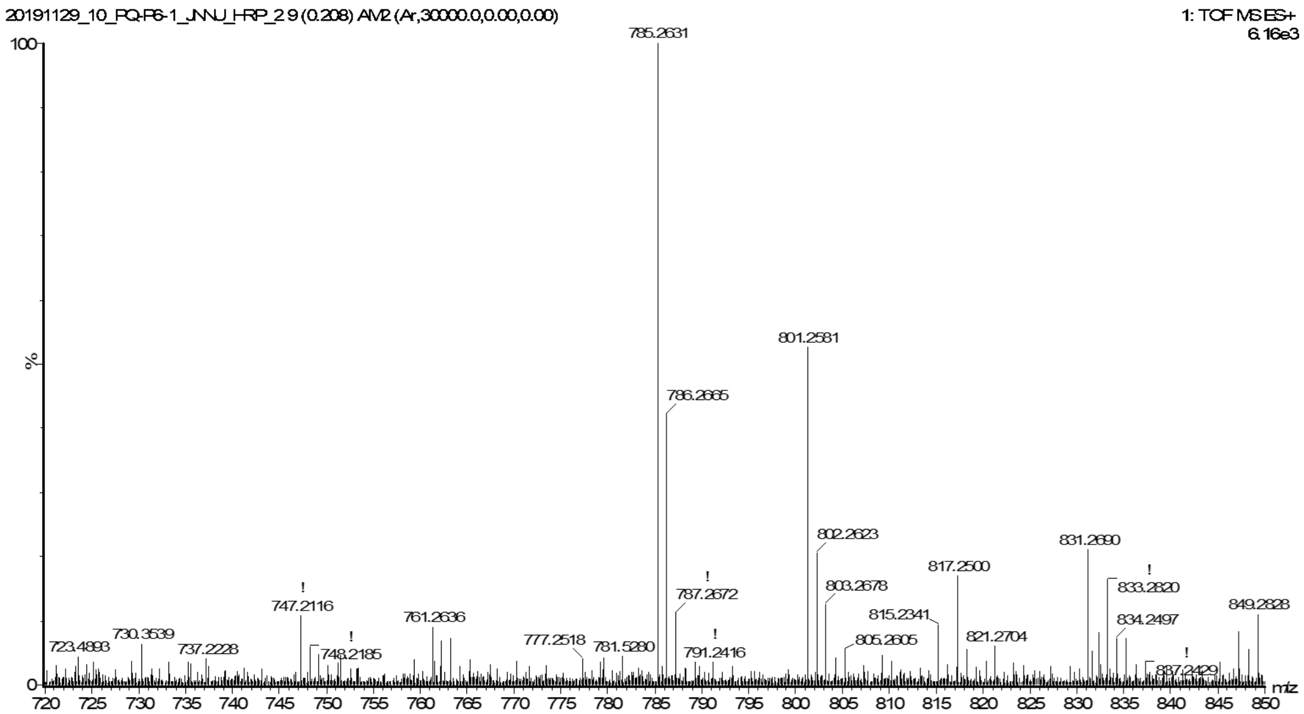

Elemental Composition Report

Single Mass Analysis
Tolerance = 5.0 PPM / DBE: min = -1.5, max = 100.0
Element prediction: Off
Number of isotope peaks used for i-FIT = 3

Monoisotopic Mass, Even Electron Ions
60 formula(e) evaluated with 2 results within limits (up to 100 closest results for each mass)
Elements Used:
C: 1-40 H: 1-50 O: 1-20 Na: 0-1

Minimum:				-1.5				
Maximum:		100.0	5.0	100.0				
Mass	Calc. Mass	mDa	PPM	DBE	i-FIT	Norm	Conf(%)	Formula
785.2631	785.2633	-0.2	-0.3	14.5	285.8	0.034	96.68	C37 H46 O17 Na
	785.2657	-2.6	-3.3	17.5	289.1	3.406	3.32	C39 H45 O17

Figure S33. HRESIMS spectrum of metabolite **8**

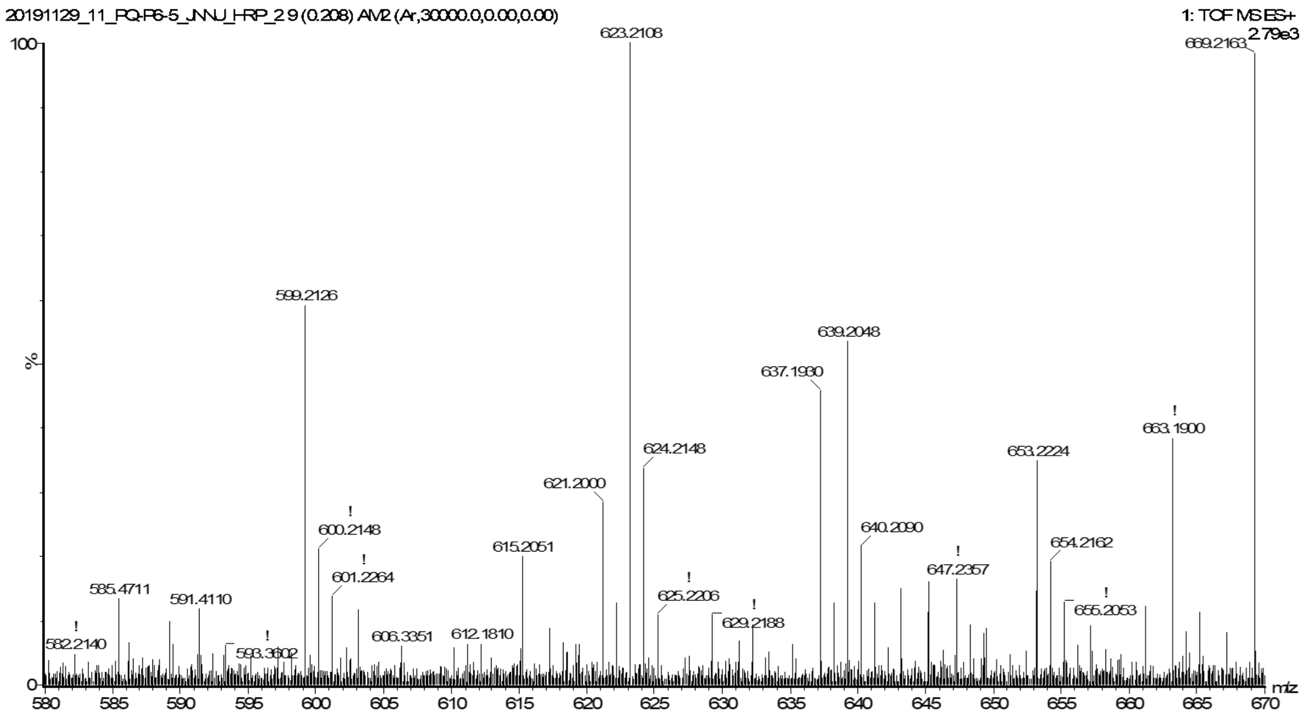

Elemental Composition Report

Single Mass Analysis
Tolerance = 5.0 PPM / DBE: min = -1.5, max = 100.0
Element prediction: Off
Number of isotope peaks used for i-FIT = 3

Monoisotopic Mass, Even Electron Ions
118 formula(e) evaluated with 2 results within limits (up to 100 closest results for each mass)
Elements Used:
C: 1-40 H: 1-50 O: 1-20 Na: 0-1

Mass	Calc. Mass	mDa	PPM	DBE	i-FIT	Norm	Conf(%)	Formula
Minimum:				-1.5				
Maximum:		100.0	5.0	100.0				
623.2108	623.2104	0.4	0.6	13.5	376.5	0.925	39.64	C31 H36 O12 Na
	623.2129	-2.1	-3.4	16.5	376.1	0.505	60.36	C33 H35 O12

Figure S34. HRESIMS spectrum of metabolite **9**

Table S1. Screening for the microorganisms that transform prenylquercetins

Microorganism name	KCTC number	Transformation capability
A. alternate	6005	-
A. coerulea	6936	+
A. fumigatus	6145	-
C. elegans var. elegans	6992	-
F. merismoides	6153	-
G. deliquescens	6173	+
G. cingulata	6075	-
M. ramanniana var. *angulispora*	6137	+
M. hiemalis	26779	+++
P. chrysogenum	6933	-
T. koningii	6042	-

Table S2. Water solubility of compounds **1-9**

Compounds	Solubility (mg/mL)	Compounds	Solubility (mg/mL)
**1**	0.013	**6**	1.978
**2**	0.063	**7**	3.635
**3**	0.020	**8**	>4
**4**	0.501	**9**	0.613
**5**	0.385	**Quercetin**	0.036
